# The long shadow of childhood cancer: a qualitative study on insurance hardship among survivors of childhood cancer

**DOI:** 10.1186/s12913-021-06543-9

**Published:** 2021-05-25

**Authors:** Manya Jerina Hendriks, Erika Harju, Katharina Roser, Marcello Ienca, Gisela Michel

**Affiliations:** 1 Department of Health Sciences and Medicine, University of Lucerne, Frohburgstrasse 3, PO Box 4466, 6002 Lucerne, Switzerland; 2 Clinical Ethics, Department of Neonatology, University Hospital Zurich, University of Zurich, Zurich, Switzerland; 3 Department of Health Science and Technology, Technical University of Zurich, Zurich, Switzerland

**Keywords:** Survivorship, Discrimination, Long-term late effects, Qualitative research, Insurance hardship, Unmet needs, Childhood cancer, Universal health coverage, Switzerland

## Abstract

**Background:**

The long-term consequences of childhood cancer have received increasing attention due to the growing number of survivors over the past decades. However, insurance hardships of survivors are mostly unknown. This study explored qualitatively, in a sample of childhood cancer survivors (CCS), (i) the experiences and needs of CCS living in Switzerland with a special focus on hardships related to insurance; and (ii) the views of insurance and law experts with experience on childhood cancer.

**Methods:**

Semi-structured interviews were conducted with 28 childhood cancer survivors and 3 experts (one legal expert, two insurance experts). Data was analysed using qualitative content analysis.

**Results:**

Three key themes emerged from the interviews with the CCS: 1) experiences with insurance, 2) perception of discrimination, and 3) needs and barriers for support. The interviewed experts provided further detailed clarification of CCS’ concerns. Our findings indicated that some CCS can move past their cancer history, while others continue to face hardships. CCS reported confusion about the opportunities and services within the social security system and most relied on their personal contacts for guidance. Finally, CCS expressed a strong need for socio-economic and legal support for social insurance questions, especially related to disability insurance.

**Conclusions:**

With the growing population of CCS, it is essential to further assess the interplay between medical and psychosocial health and socio-economic hardship. Supportive psychosocial services should aim to ameliorate insurance hardships. Better understanding of the relationship between childhood cancer and insurance hardships during survivorship will inform efforts to improve long-term financial security and health outcomes for survivors. We call for the public, lawmakers, researchers, insurers, and patient organizations to come together and discuss future perspectives to avoid the risk of discrimination for cancer survivors.

## Introduction

The long-term consequences of childhood cancer have received increasing attention due to the growing number of survivors over the past decades [1, 2]. In particular, the life-long risk of adverse health and psychosocial effects for childhood cancer survivors (CCS) have been extensively described [3–5]. Only recently, studies have focused on the socio-economic hardships of cancer survivors and their families [6–12].

CCS report higher out-of-pocket medical expenses [13, 14], are more often uninsured [6, 13], face difficulties in obtaining life insurance [15–17], and have a higher probability of requiring social security or disability benefits [18]. These kinds of hardship are shown to cause or potentially exacerbate physical and psychological harms [6, 14, 19], including anxiety, stress, and impaired sleep [20]. Conversely, some physical, neurocognitive, and psychological late effects may lead to hardship such as low income and financial difficulties [7, 21–23].

The challenges faced by CCS regarding access to insurance and social security have not been extensively examined [15, 24]. Moreover, the bulk of this research has focused on CCS’ experiences in the United States where, in the absence of universal health care, CCS may be more vulnerable to such hardships [6, 13, 25, 26]. Correspondingly, these findings cannot be generalized to countries with other social security systems in place. In Switzerland, for example, every resident must be affiliated to and is covered by basic health insurance [27–29]. Furthermore, compulsory disability insurance (DI) aims to guarantee the basic needs of persons who have become disabled, by paying disability benefits and/or by providing rehabilitation measures [30–32]. In addition to basic health insurance and DI, there are optional private insurance schemes such as supplementary health insurance (which may for instance encompass alternative medicine or psychotherapy) and primary private insurance (e.g. life insurance) [33, 34]. In Switzerland the employer partly pays accident insurance. Meaning that individuals without employment have to finance this themselves. This poses an additional hardship to CCS who are more likely to be unemployed or face difficulties to entering the workforce [10]. Navigating through this complex system can be challenging for CSS. Although some support services exist, such as *Procap* and *Kinderkrebshilfe* Schweiz [35], most of them are either not specialized in childhood cancer or the social security system and often CCS and their families are not aware of these services [36].

As a country with a long history of high healthcare costs and universal coverage, Switzerland offers a valuable environment to explore the experiences and needs of CCS regarding several forms of insurance such as health, disability, and private insurance. With the growing number of CCS due to improved treatment, recognizing the socio-economic hardships of CCS is crucial. Effective strategies that meet CCS’ needs are central to tackle financial barriers and challenges that CCS face during their long-term survivorship. Currently, follow-up care for CCS is reportedly lacking psychosocial support at many pediatric oncology centers despite its health economic relevance [36–38]. Therefore, this study aimed to qualitatively assess the experiences and needs of CCS living in Switzerland with a special focus on hardships related to insurance. This assessment is further complemented by an exploration of the views of a sample of insurance and law experts with experience on childhood cancer.

## Method

### Study design

This study builds on a larger mixed-methods project on the impact of cancer and unmet needs of CCS during survivorship, which combined a quantitative and qualitative design [38]. In the current study, we used a qualitative research approach [39] and conducted semi-structured interviews with a subset of CCS from a cross-sectional survey and insurance and legal experts.

### Sample and procedure

CCS had been identified through Childhood Cancer Switzerland, the umbrella organization of institutions in pediatric oncology in Switzerland. Participants were eligible if they were aged ≥ 18 years at time of study, diagnosed with cancer ≤ 18 years of age, completed treatment ≥ 2 years before study, were Swiss residents, and spoke German or English.

 Childhood Cancer Switzerland sent an e-mail invitation to participate in the cross-sectional survey to all registered survivors (*n* = 132). After two months, a reminder was sent to non-responders. Additional participants were invited through an open electronic link that was circulated among Swiss CCS’ networks on social media platforms such as Twitter, and survivors’ messaging services on Whatsapp as well as survivor physical meetings. Participants completed the cross-sectional survey during which they were invited to participate in an interview to obtain a more nuanced understanding of their experiences, preferences and needs regarding their long-term survivorship. After initial analysis of interviews with CCS, we also recruited three experts (one legal expert, two insurance experts) by means of purposive sampling. Experts were identified through Childhood Cancer Switzerland to explore the challenges and barriers to support services for insurance hardship raised by CCS in the interviews from experts’ view.

### Ethical approval

The study was approved by the Ethics Committee Northwest and Central Switzerland (Study-ID: EKNZ 2017-01758). Before the interviews, participants received written and oral information about the study. They were ensured anonymity and provided informed consent. The project was conducted in line with the Helsinki declaration.

### Data collection

 Survey participants who showed interest in participating in the interviews shared their contact information with the study team. Interviews were scheduled at a place of their choice (the participants’ home, workplace, or private meeting room at a Swiss University). The interviews were carried out until theoretical saturation was achieved [39]. All interviews were conducted between November 2017 and February 2019. Data were collected using a semi-structured interview guide developed by the study investigators (MH and GM). The interview guide focused on CCS’ experiences of their childhood cancer and survivorship [38]. Some CCS showed photographs, diaries, and other memories of their cancer and survivorship experience. CCS’ experiences further informed the interview guides for expert interviews, which entailed open questions related to the Swiss healthcare system. The audio-recorded interviews lasted between 39 and 117 min (89 min on average) and were transcribed verbatim by a transcriptionist.

### Data analysis

Analysis was conducted in the form of qualitative content analysis. Two members of the research team (MJH and EH) reviewed the transcripts to identify emerging themes and developed a coding structure guided by Kuckartz’s approach to content analysis [40–42]. This approach integrates elements from grounded theory such as theoretical memos and iteration to generate not only descriptive results but also conceptual models of the topics under study. First, the initial coding structure was developed based on our research question, interview guide, and reviewed literature (MH, EH). Second, preliminary codes were generated through systematic coding of the data by first and second author. Then identified codes were reviewed and refined. Consensus for coding was reached dialogically among the two authors. All transcripts were re-coded at the end of the process using the finalized coding structure. Qualitative data organization and aggregation was facilitated by ATLAS.ti 8.3.

## Results

### Sample characteristics

Of the 69 respondents of the previously administered survey [38], 51 expressed interest to further participate in the interview study. Subsequently approached by the study team, 21 individuals did not reply, one declined participation, and two could not find the time to make an appointment. We conducted interviews with the remaining 28 participants (response rate 55 %). Among them, the most frequently reported diagnosis was leukemia (10/28). Survivors had completed treatment on average 19 years prior. Of the 28 participants, 68 % were female (mean age at study: 31 years; mean age at diagnosis: 9 years; Table 1). All interviewed experts (one legal, two insurance experts) had >25 years of professional experience (Table 1).

**Table 1:** Sample description of participants

**Childhood cancer survivors**	
**Total**	*N* = 28 (100%)
**Sociodemographic characteristics**	
Gender	
Male	9 (32.1)
Female	19 (67.9)
Age at study	
≤25years	11 (39.3)
26-30 years	5 (17.9)
31-35 years	4 (14.3)
>35 years	8 (28.6)
Nationality	
Swiss	24 (85.7)
Swiss and other nationality	4 (14.3)
Currently in a relationship	
Yes	6 (21.4)
No	22 (78.6)
Children	
Yes	6 (21.4)
No	22 (78.6)
Education	
Compulsory schooling	4 (14.3)
Vocational training	13 (46.4)
Upper secondary	6 (21.4)
University degree	5 (17.9)
Employment status	
Employed	24 (85.7)
Unemployed	4 (14.3)
**Clinical characteristics**	
Diagnosis	
Leukemia	10 (35.7)
Lymphoma	5 (17.9)
CNS tumor	3 (10.7)
Other^a^	10 (35.7)
Age at diagnosis	
0-5 years	7 (25.0)
6-11 years	10 (35.7)
12-18 years	11 (39.3)
Treatment	
Surgery only or chemotherapy^b^	15 (53.6)
Radiation^c^	10 (35.7)
Bone marrow transplantation^d^	3 (10.7)
Time since end of treatment^e^	
≤5 years	3 (11.1)
6-15 years	8 (29.6)
16-25 years	6 (22.2)
>25 years	10 (37.0)
Late effects	
Yes	18 (64.3)
No	10 (35.7)
Type of late effects^e^	
Physical	12 (75.0)
Physical and psychological	4 (25.0)
Follow-up attendance	
Yes	15 (53.6)
No, completed	13 (46.4)
Relapse	
Yes	7 (25.0)
No	21 (75.0)
Second malignancy	
Yes	6 (21.4)
No	22 (78.6)
**Mean (years, range)**	
Age at study	31.4 (18-55)
Age at diagnosis	9.3 (0.5-16)
Time since end of treatment	19.1 (2-38)
**Experts**	
**Total**	*N* = 3 (100%)
**Sociodemographic characteristics**	
Gender	
Male	2 (66.6)
Female	1 (33.3)
**Mean (years, range)**	
Age at study	51.6 (41-56)
Working experience	26.3 (21-33)

^a^Other includes neuroblastoma, renal tumor, bone tumor, soft tissue sarcoma, thyroid cancer, germ cell cancer and histiocytosis

^b^Has not included radiation

^c^May have included surgery and/or chemotherapy

^d^May have included surgery, chemotherapy and/or radiation

^e^Missing values

Overall, three key themes emerged from the interviews with the CCS: 1) experiences with insurance, 2) perception of discrimination, and 3) needs and barriers for support. The interviewed experts provided further detailed clarification of CCS’ concerns, which are grouped to match the themes. In the following, we describe each theme in detail.

### Experiences regarding insurance

CCS expressed concerns about the many different facets of insurance, ranging from basic health insurance to DI and private insurance (Table 2). As one CCS phrased it; “*What I think has stuck with me most of all are not the physical aspects but the whole thing with the insurances.*”

**Table 2:** Selected quotes on insurance

	**Demographics**	**Quote**
**Basic health insurance**	Male survivor, >25 years since treatment (2M)	Once, I had to make my scar more beautiful, and the […] health insurance would only finance the cheapest [treatments], but otherwise I never had problems, I just could not change the supplementary [health] insurance, but otherwise they [health insurance] were very accommodating.
	Female survivor, 6-15 years since treatment (5F)	A while ago, I had broken a tooth because of the radiation. [...] They wanted to do a dental crown, which costs a lot of money, and the health insurance said they won't pay. [...] Because they say it can't be because of the radiation. Now I am covered by the legal insurance of my parents, with a lawyer backing it up, now we are suing the health insurance so that they have to pay for the tooth. I have all the documents from the radiation – from the dentist, my ENT specialist and from the radiation. It really happened and it's not my fault. It was actually expected that these problems would already arise 5 years ago, because they often break after 5 years, but I took care of my teeth too well. Brushing my teeth too well. And seeing as it is only happening now, in their opinion, it is too late now. In their point of view there can’t be any connection because it is too long ago. And well my ENT specialist says, because she took such good care of it, it's 5 years later; "*Don't be stupid, just pay for it*." Yes, yes, and by law they should pay. Health insurance article 19, ((laughs)) I have learned that, yes, I also had a meeting with the lawyer who is looking into it, and he said they have to pay.
**Disability insurance**	Female survivor, 16-25 years since treatment (16F)	[after finding a late effect] my doctor also thought that we should clarify about applying for DI and so on, and I said right away that I didn't want to, because there are enough DI recipients who shouldn't be, where it's actually not justified [...] I don't feel as if I'm entitled to demand something like that, because I can work 100%, I've been in this profession for two and a half years now after my apprenticeship, and yes, I never had the feeling that I needed or wanted it.
	Female survivor, 6-15 years since treatment (27F)	I know I was once told I could get DI if I wanted to. I missed jumping on that train because I just thought, no, I'm doing very well. There are people who really need it, who have to get DI and now I have been thinking, why didn't I do that back then and just seize this opportunity. Not to exploit, not at all, but more like, then I could have looked more after, a little better after my health. But now it's like this and it's going well, so now I have the feeling that I don't need to apply for DI, but maybe I could take better care of myself, because then it would look better financially.
**Private insurance**	Female survivor, >25 years since treatment (22F)	But I realize now, just like that, other life insurance policies or even occupational insurance or [supplementary] health insurance, which I have taken out because of my job, that there are simply restrictions everywhere. Even the best insurance agent can't fix more than that. They just say like, […] if you have this and that, then they don't pay it, they only pay for broken legs, broken arms, but if I have anything like that with blood, they don't pay anything. Interviewer: even if it was how many years ago, actually? Participant: Yes it is stupid, it was at the age of five and a half and so 31 years [later]. No, they do not pay it.
	Male survivor, 6-15 years since treatment (4M)	I mean she [insurance broker] knew that I had had this, but she also knew that I am healthy and that I will not have a relapse. Well they say, after ten years, they say, you are healthy so you are completely cured. In fact, you will not relapse anymore and because of that you can indicate it [disease history] without hesitation. Then you no longer have to declare it […] and you can change [supplementary health insurance] freely.

With regard to basic health insurance, most CCS were satisfied with the coverage. However, some CCS also mentioned that they had to actively invest time and energy into making sure they were reimbursed for their healthcare costs. CCS, who reported difficulties with their basic health insurance, similarly pointed towards difficulties with reimbursements. This encompassed novel treatments such as fertility costs or rehabilitation treatment abroad and included costs caused by dental late effects.

CCS described different concerns relating to DI, in which CCS with late effects or disability can request to reduce working hours. CCS who received DI or were in the process of acquiring DI described it as a challenging procedure. One CCS reported having to “*muddle through*” the entire process for two and a half years. Others reported they had to explain a lot and questioned whether staff assigned to their application could “*really understand what the problem is of the whole thing [cancer and its late effects]*”. One CCS described how he went through a challenging time in applying for DI. He experienced the questions posed by medical experts in charge of approving his DI application as very stressful. In addition, the CCS mentioned he was not allowed to have anyone present who could support him during the questioning. He stated that in getting his medical approval for DI, he had to go through “*3 hours of almost psycho-terror*”. Or as another CCS reported: “*When you are really unstable and mentally not quite there […]. Then you are helpless, lost - really simply at their mercy.*”

While some CCS were in the process of requesting DI, others were reluctant to apply despite potentially being qualified. Those who were currently reluctant expressed either a negative attitude toward being a DI-recipient or wanted to resume their normal life. Additionally, some CCS who had been reluctant earlier, and refrained from applying for DI, did not know how to apply for DI now, and some faced difficulties with their application. The interviewed experts further explained this:

*Especially in the labor market it leads to a situation in which if a person notices that they cannot work 100 % [fulltime position], because they just can’t cope, people simply say then “I will reduce 20 % of my workload”, but they don’t report it [to the DI] and then they have voluntarily reduced. […] and somehow 10 years later when they reduce again from 80 to 60 % they declare [to the DI] that this is now because of demonstrable medical late effects of cancer. Only then are they considered 20 % disabled because before that they have ‘voluntarily’ reduced their workload [from 100 % to 80 %]. In reality, they would be 40 % disabled, but because they did not declare the first 20 % years before, because they did not want to admit they need DI, for understandable reasons, they punish themselves because otherwise they would have DI in addition to a 60 % income. If you then perhaps still have family obligations, it is then simply financially tight. And then there is the question again, do you still have to manage working 80 % pensum, because they need it financially. Then there is a vicious circle. That is what we still experience very often.* – Legal expert at a patient organization, > 25years of working experience.

The three experts mentioned the importance of providing appropriate advice to all CCS who, due to their late effects, may have to reduce working hours in the future and therefore apply for DI. For this reason, CCS must be adequately informed about the measures to be taken, which requires legal information.

In the context of private insurance, CCS reported difficulties with supplementary health insurance or life insurance. CCS who did not already have supplementary health insurance before childhood cancer were only eligible for insurance that was not related to their cancer diagnosis or its late effects. In situations where they had supplementary insurance prior to their childhood cancer, they were often no longer able to make changes to their insurance policy. CCS reported similar concerns with their life insurance either it would not make financial sense; premiums would be higher and/or cancer would be excluded. Only few CCS reported no problems at all in getting private insurance despite their childhood cancer history. One CCS was not requested to report his medical history, although this is a standard and legal procedure of insurance companies for supplementary health and life insurance. According to him, his “*insurance broker at the time, [who] also had had cancer and everything. Yeah, […] he said you should let that [the cancer] rest at some point*.” Another CCS described how he was able to get supplementary health insurance 10 years after being curatively treated and while attending follow-up care.

### Perception towards discrimination

In some CCS, their social and health insurance experiences evoked feelings of “*discrimination*” and “*unfairness*”. CCS claimed that because their cancer history is not a lifestyle choice, when their health insurance excludes them from supplementary insurance, it feels as if they are punished for something that is beyond their accountability. One CCS mentioned that “*if that happens on the basis of the illness, […] that’s not fair, because in most cases you cannot help it.*” Another CCS said that “*Leukemia, that’s something that can happen to anyone […], but that’s the way the system works, I don’t think it’s any use getting worked up about it*.” Some CCS argued that it would be different if people are excluded who can be held accountable for their behavior (e.g. due to smoking or risky sport behaviors).

*Sure if someone smokes for 40 years and then suddenly, yes […]. But some people have been smoking for 40 years and they don’t have such effects, but that’s the way it is with insurance companies. If you do risky sports, at some point someone found out what risky sports are. Of course, I can never have a riding accident if I don’t go riding, simple, yes. But in case of illness, nobody knows that. That’s why I think it’s not good to be classified that way. And somehow you get a feeling that you are less valuable because you had that illness, so yes, not nice – Male survivor, > 25 years since treatment (19 M)*.

### Need and barriers for support

CCS expressed a strong need for legal support for themselves and their families or parents whose children are diagnosed with cancer. CCS further specifically wished for a point of contact to turn to for legal questions and/or concerns regarding insurance such as health, disability, and private insurance. CCS acknowledged that addressing these topics or concerns would go beyond healthcare professionals’ expertise, but they wished someone would be able to guide them through insurance issues.

Most CCS described how, in the end, they got legal help through their personal contacts (i.e. parents, spouse or social network). One CCS explained how her parents’ legal insurance had allowed her to contest the unwillingness of her basic health insurance to cover a dental procedure which became necessary due to a late effect. Without the legal insurance of her parents, she would have had to pay the procedure herself. In addition, without the support of a legal service the problems faced by CCS are extremely time consuming. CCS shared stories on how they would not contest some denied reimbursement claims since they “*do not want to get into the whole thing and get into such a feud.*”

*I have to advise everyone to get a lawyer. […] I think that, if only there was a place where I could have gotten specific advice on this […], or a lawyer specialized in this. […] Now in the end I asked my godparents; they are both lawyers. Now after this I have to say: As long as someone who had cancer can still prove what their late effects are, and that is why they cannot work fulltime, and they can really prove it comes from the, for example, chemotherapy […] I have to recommend everyone to get a lawyer. – Male survivor, >25 years since treatment (3M)*

Finally, the interviewed experts emphasized the increasing need for interdisciplinary support on socio-economic aspects during CCS’ follow-up. Although they agreed that their recommendations would not substitute any medical advice, they argued that, for the same reason, healthcare professionals should not make “*legal, social insurance, or employment law ‘diagnoses’*”. This was partly motivated by the fact that, in some circumstances, such extra-medical advice by health professionals led to inaccurate information provided to the CCS. Experts reported that it can be incredibly difficult to correct legal misinformation when it is provided to the CCS by healthcare professionals;

*When the client [CCS] tells me, yes, but the doctor has said that it [the second wheelchair] will be reimbursed. Then I say the doctor can’t promise this as he’s not competent to do so. It’s just going to confuse the client. Should they believe the doctor or the lawyer because one is ‘lying’, I find this a pity. – Legal expert at a patient organization, > 25years of working experience*.

Experts further mentioned the lack of a well-functioning network between healthcare professionals and interdisciplinary staff (i.e. social workers, (social) insurance and/or legal experts) as a primary barrier to appropriately refer CCS with needs to available support services. However, experts reported that recently both, experts and heads of pediatric oncology departments, are recognizing the need for interdisciplinary collaboration. One center has started to invite a legal expert to a monthly consultation hour for families, patients and survivors. With better collaboration between experts and healthcare professionals, experts are convinced that it will not necessarily increase demand. Instead it will enable better advice, less miscommunication for those CCS vulnerable to socio-economic hardship, and more awareness among healthcare professionals regarding the socio-economic or legal concerns of CCS and families.

## Discussion

This qualitative study has revealed several hardships faced by CCS when managing their survivorship trajectory and accessing insurance coverage. Previous research has shown that while late effects can exacerbate hardships, the hardships themselves can also cause physical and psychosocial harm [21]. Indeed, CCS in our study have reported that hardships related to insurance are a frequent cause of stress, need time-consuming effort, and require interdisciplinary support. These findings suggest that insurance providers and regulators should devote more attention to preventing and mitigating such hardships. Although the Swiss social security system can alleviate the financial burdens for some CCS through, for example, disability benefits [30, 34], structural challenges and social restraint might hinder access to these services.

### Adequate information and collaboration to combat information gaps

From a societal perspective, our study raises the question of adequately informing CCS and their families about DI, since many CCS expressed a negative attitude towards DI and described it as something to be avoided. This is nothing new. It is known that applying for sick-related benefits is often connected to feelings of stigma or insecurity due to the strong desire to be financially self-sufficient [43]. In addition, studies have shown that making insurance claims involves extensive workload and psychological stress [32, 44], which might hinder CCS from making such claims. This stands in stark contrast to how DI recipients are often portrayed in media and social discourse [45]. While recognized in qualitative research [32, 46], such negative accounts of patients’ lived experience with DI are largely absent from public discourse. Albeit it is important to note that CCS with severe neurocognitive effects of treatment whom require DI from the time of treatment completion (e.g. brain tumor survivors) are more likely to enroll or to be enrolled by their parents and caregivers [47, 48]. However, our study indicates that CCS who could benefit from some form of DI, might nonetheless refrain from doing so due to the negative attitude towards DI. This is confirmed by studies showing that many survivors, when categorized as ‘cured’ from cancer, wish to live everyday life without being reminded of their past and marked with a stigma [15]. Therefore, CCS’ hesitancy to apply for DI might be related to defense mechanisms to reduce anxiety arising from psychologically harmful reminiscences [32]. Most importantly, such attitudes may be strongly intertwined with questions of personal identity, in particular to the self-perception of the CSS and their transition from a patient-based to a survivor-based perspective. Although much has been written about this identity paradox when moving from a “cancer identity” to a “survivorship identity” [49], little is known about how CCS’ may tend to re-negotiate their identity when placing themselves on the (dis)ability scale for their occupation.

From a structural perspective, this study shows that CCS in Switzerland may lack adequate information, knowledge, and support regarding insurance issues during their survivorship trajectory. These findings suggest that more legal support is needed for CCS and their families. This need for information on legal and insurance issues has recently been recognized by childhood cancer patient organizations in Switzerland, which have started to offer more services, e.g. information-weekends on this topic for parents of CCS [50]. An improved development of such services is required as is building awareness among healthcare professionals on socio-economic or legal concerns and the benefits of interdisciplinary exchange and networking with legal experts and social workers. In addition, better collaboration between these actors could support and guide CCS and their families through the administrative and insurance hardships they may face now or in the future. Although medical and social issues are understandably at the forefront of concern for CCS and their families, it is crucial that healthcare professionals raise the topic of socio-economic and insurance issues to CCS and their parents early on. By doing so, CCS and their parents will be able to gather information on socio-economic topics and thereby request and receive support early into survivorship. Intervention and legal support from specialists with knowledge on childhood cancer and legal proceedings can further prevent misunderstandings between healthcare professionals and CCS. It will additionally help to prevent CCS from making preventable mistakes in the administrative and insurance aspects of the social security system [51].

### The right to be forgotten

Furthermore, as many CCS in our study mentioned being excluded – in some way – from private insurance the scope and justification of insurance exclusions require greater attention. In Switzerland, apart from basic insurance, it is generally obligatory for insurance candidates to declare their medical history to most private insurances (such a supplementary health insurance or life insurance). This includes someone’s cancer history. Based on the medical history, the insurer is legally entitled to impose higher premiums (increasing the cost of insurance), limit the insurance policy by excluding some risks (including late effects from cancer and its treatment), or even refuse insurance. Failure to disclose the complete medical history can result in cancellation of the insurance policy, even if certain experienced health-conditions are entirely unrelated to cancer, and could potentially result in prosecution for fraud. In Switzerland, this dynamic has led to several legal proceedings for discrimination initiated by citizens against insurers, none of which has been favorably judged by a court [52]. Although it has not yet been recognized by current case law, there is strong evidence that current insurance policies have considerable potential for discrimination on the grounds of illness or disability [53–55]. Our findings corroborate this evidence at the level of the CCS first-person perspective and lived experience, revealing that many CCS felt discriminated against for something that happened a long time ago, and without their “fault”. Further, most CCS reported that their private insurance policies excluded, inter alia, cancer relapses or late effects. This problem is not exclusively restricted to the Swiss social security system. In 2018, the European organization “Youth Cancer Europe” named financial discrimination as one of the five key challenges needed to be addressed on a national and European policy level [16]. More and more studies show that CCS are excluded from mortgages and private insurances such as life insurance, supplementary health insurance or travel insurance [16, 56–58]. This results in many CCS being discriminated against (as recipients of unequal treatment) either via denied insurance or by having to pay higher premiums than people without chronic health-conditions [58]. There is growing agreement among civil society organizations (e.g. patient alliances) and regulators that current policies need to be amended to mitigate the risk of discrimination for cancer patients in the insurance domain. For example, the European Cancer Patient Coalition has advocated the view that, once the cure of cancer is declared, survivors should have the right to live their lives without being discriminated for their cancer history [59]. Irrespective, of how they are doing, compared to other people of similar age and socio-demographic characteristics with no cancer history.

In recent years, this shift of perspective has gained momentum among policy makers in several European countries. In 2016, France first introduced the “Right to be forgotten” law, which grants cancer survivors the legal right not to disclose their cancer diagnosis to insurers after a period of 10 years after end of treatment, or for cancers occurring before the age of 18 years, or 5 years after end of treatment [59, 60]. With the implementation of the European Union’s General Data Protection Regulation (GDPR), the right to be forgotten has been enshrined in federal data protection regulation, enabling individuals to request the deletion or removal of specific personal data (including health data) where there is no compelling reason for its continued processing (Art. 17) [61]. Thereby the GDPR provides the lawful basis and legal framework for enabling CCS to have their data erased or rectified by the data controller (e.g. the insurance provider). Also, at the European Union level, the right to be forgotten for cancer patients has been included in the ‘Europe’s Beating Cancer Plan’ revealed by the European Commission in early 2020 [62]. According to the GDPR, however, a CCS’ right to be forgotten can be overridden by a health insurer’s right to process someone’s data when the data represents information that serves the public interest or is deemed necessary for statistical or public health purposes [61].

For this reason, national derogations are necessary to determine the conditions of applicability of the right to be forgotten, leading countries like France to make explicitly inclusive provisions for CCS in their national legislation. Belgium, The Netherlands, and Luxembourg have since followed the French approach and adopted similar national laws to protect cancer survivors [63]. Besides data protection, attempts to reduce discrimination have also occurred in the context of the health law. For example, the 2010 ‘Affordable Care Act’ in the United States entails provisions forcing insurers to accept all applicants without charging based on pre-existing conditions [64].

### Limitations and strengths

Our study has several limitations and strengths. One limitation is the risk of self-selection since some CCS may have been more reluctant to participate in the study [39], a typical limitation of qualitative approaches. Furthermore, despite self-reported unmet needs, CCS might have received additional psychosocial or socio-economic support, but forgotten about it, or support was offered to their parents [65]. Second, it is possible CCS who have had negative experiences could have been more eager to voice their issues and concerns. Third, due to the study setting and the inherent diversity of different national insurance systems, the findings of our study may not be fully generalizable to other countries. However, two mitigating factors must be considered. First, statistic-probabilistic generalizability is not inherent in the scope of qualitative research — which is focused on gaining a deeper understanding and uncovering deep-rooted explanations of phenomena. Second, statistical generalizability does not subsume generalizability tout court, with research showing different types of generalization such as transferability and analytical generalizability [66].

To our knowledge, this is one of the first studies to investigate the impact of cancer survivorship within the Swiss healthcare system. In addition, the Swiss healthcare system shares common features with multiple international healthcare regimes, such as the French and Italian ones. Among those, common features are, being universal in character and entailing a basic insurance for all. However, since there are no free state-provided health services and private insurance is compulsory for all residents, the Swiss system also shares commonalities with healthcare systems largely operated by the private sector such as the United States. For these reasons, we believe the findings of our study can provide analytically valuable and transferable information on the insurance-related hardships faced by CCS. A further strength of our study was our effort to enable CCS with severe late effects or impairments to meet at a most comfortable location (at work, university, library, home), hence facilitating their participation. In addition, the diversity of our sample of CCS enables us to gain a multifaceted picture of insurance hardship for CCS in Switzerland. Finally, the combined perspectives of CCS and experts enriches the results of our study.

## Conclusion

Our findings seem to indicate that some survivors can move past their cancer history, while others continue to face hardships. More importantly, confusion exists about the opportunities and services within the social security system and most CCS relied on their personal contacts for guidance. Hence, CCS expressed a strong need for socio-economic and legal support for social insurance questions, especially related to DI.

With the growing population of CCS, it is essential to further assess the interplay between medical and psychosocial health and socio-economic hardship. Supportive psychosocial services should aim to ameliorate insurance hardships. Better understanding of the relationship between childhood cancer and insurance hardship during survivorship will inform efforts to improve long-term financial security and health outcomes for survivors [67, 68]. Further research on insurance issues concerning CCS and their parents, while contextualizing the Swiss social security system, can help to establish national interventions that promote family adaptation, problem solving, coping, and resilience. Furthermore, it is necessary to mitigate specific mechanisms that create financial or other strain not only on families having experienced childhood cancer but also families with chronically ill children. In doing so, the perspectives of legal, healthcare, and insurance experts are essential to guide solutions to the challenges and barriers reported by CCS and their families and other vulnerable populations.

Finally, based on our study’s findings, we endorse policy reforms as the “Right to be forgotten” and call for the public, lawmakers, researchers, insurers, and patient organizations to come together and discuss future perspectives to avoid the risk of discrimination for cancer survivors. Such public debate is critical to ensure “the reintegration of survivors to normal social roles and activities without discrimination” [69].

## Data Availability

The datasets generated and/or analyzed during the current study are not publicly available due to risk of participant privacy being compromised but datasets may be available from the corresponding author on reasonable request and with permission from the responsible Ethics Committee.

## Abbreviations

CCS: Childhood Cancer Survivors
DI: Disability Insurance
GDPR: General Data Protection Regulation
